# Pronounced antibody elevation after SARS‐CoV‐2 BNT162b2 mRNA booster vaccination in nursing home residents

**DOI:** 10.1111/irv.13030

**Published:** 2022-08-12

**Authors:** Yong Chong, Takeyuki Goto, Naoki Tani, Akiko Yonekawa, Hideyuki Ikematsu, Nobuyuki Shimono, Yosuke Tanaka, Koichi Akashi

**Affiliations:** ^1^ Medicine and Biosystemic Science Kyushu University Graduate School of Medical Sciences (The First Department of Internal Medicine) Fukuoka Japan; ^2^ Japan Physicians Association Tokyo Japan; ^3^ Center for the Study of Global Infection Kyushu University Hospital Fukuoka Japan; ^4^ Medical Corporation SOUSEIKAI, Kanenokuma Hospital Fukuoka Japan

**Keywords:** antibody response, booster vaccination, COVID‐19, nursing home residents, SARS‐CoV‐2

## Abstract

**Background:**

Infection control during COVID‐19 outbreaks in nursing facilities is a critical public health issue. Antibody responses before and after the third (booster) dose of SARS‐CoV‐2 vaccination in nursing home residents have not been fully characterized.

**Methods:**

This study included 117 individuals: 54 nursing home residents (mean age, 83.8 years; 39 SARS‐CoV‐2‐naive and 15 previously infected) and 63 healthcare workers (mean age, 45.8 years; 32 SARS‐CoV‐2‐naive and 31 previously infected). Anti‐spike (receptor‐binding domain [RBD]) and anti‐nucleocapsid antibody responses to BNT162b2 mRNA vaccination and their related factors were evaluated using pre‐ (shortly and 6 months after the second dose) and post‐booster vaccination samples.

**Results:**

The median anti‐spike (RBD) IgG level in SARS‐CoV‐2‐naive residents 6 months after the second dose was the lowest among the four groups, with a decreasing rate of over 90%. The median rate of increase before and after the third dose in SARS‐CoV‐2‐naive residents was significantly higher than that in SARS‐CoV‐2‐naive healthcare workers (64.1‐ vs. 37.0‐fold, *P* = 0.003), with the highest level among the groups. The IgG ratio of SARS‐CoV‐2‐naive residents to healthcare workers after the second and third doses changed from one‐fifth (20%) to one‐half (50%). The rate of increase after the third dose in previously infected individuals was three‐ to fourfold, regardless of residents or healthcare workers.

**Conclusions:**

Advanced aged nursing home residents, poor responders in the initial SARS‐CoV‐2 vaccine series, could obtain sufficient antibody responses with the additional booster dose, despite more than 6 months after the second.

## 1 INTRODUCTION

The coronavirus disease (COVID‐19) pandemic, caused by severe acute respiratory syndrome coronavirus 2 (SARS‐CoV‐2), has severely affected long‐term care facility residents due to their advanced age, frailty, and co‐morbidities.^1^ Although vaccination against SARS‐CoV‐2 has proven to show high clinical efficacy, diminishing effectiveness over time after vaccination has been shown in nursing home residents and the general population.^2^, ^3^ A reduction in specific antibody responses to SARS‐CoV‐2 vaccination has been shown to correlate with an increased risk of breakthrough infections.^4^, ^5^ In such a situation, the third (booster) dose of SARS‐CoV‐2 vaccines is currently in progress, and the recovery of both post‐booster antibody responses and vaccine efficacy has been shown.^6^, ^7^ However, the detailed kinetics of antibody levels after the post‐second and third doses in nursing home residents have not been fully characterized, although a few studies have been conducted on this topic.^8^, ^9^, ^10^

Our previous study at a nursing facility in Japan where a COVID‐19 outbreak was experienced showed that the specific antibody responses after the initial BNT162b2 mRNA vaccination in SARS‐CoV‐2‐naive nursing home residents were markedly lower than those in healthcare workers.^11^, ^12^ In contrast, previously infected residents obtained rapid and robust antibody responses comparable with healthcare workers.^12^ Here, we evaluated the antibody responses shortly after the second, 6 months after the second, and shortly after the third dose in the same cohort at the facility, including SARS‐CoV‐2‐naive and previously infected residents and healthcare workers. Detailed information on the immunogenicity of a series of SARS‐CoV‐2 vaccinations in nursing home residents could be useful for maintaining effective control measures for future COVID‐19 outbreaks in primary healthcare facilities.

## 2 METHODS

### 2.1 Participants and sample collection

This study was conducted as a serological follow‐up evaluation after reporting specific antibody responses after the initial SARS‐CoV‐2 vaccine series at a nursing home with an outbreak in April, 2020.^12^ In the same cohort, individuals whose samples were obtained 6 months after the second dose and shortly after the third dose were included. The period and diagnosis of infection in previously infected nursing home residents and healthcare workers are shown in a previous report.^12^ The initial BNT162b2 mRNA vaccination was performed twice at a 21‐day interval from May to July, 2021. The third (booster) vaccination program was conducted from January to February, 2022 using the same BNT162b2 vaccine, resulting in an interval of approximately 7 months between the second and third doses. Serum samples shortly after each dose and 6 months after the second dose were collected on the scheduled days 21 and 180, respectively. Sampling 21 days after dose was referred to as “shortly after dose.” Clinical data, including information on underlying diseases (Table 1), were collected from medical records and questionnaires. Serological testing and accompanying clinical data collection were based on the informed consent obtained from participants or family members and approval by the Ethics Committee of the Hara‐doi Hospital.

**TABLE 1:** Demographic characteristics in healthcare workers and nursing home residents

	SARS‐CoV‐2‐naive	Previously infected	SARS‐CoV‐2‐naive	Previously infected
Characteristic	HCW	HCW	NHR	NHR
Total number	32	31	39	15
Gender, male	15 (46.9)	10 (32.6)	13 (33.3)	6 (40.0)
Age, mean years ± SD (range)	48.6 ± 12.3 (27–69)	44.8 ± 11.6 (21–65)	84.5 ± 7.0 (64–96)	85.5 ± 9.9 (62–100)
Chronic underlying disease				
No disease	28 (87.5)	23 (74.2)	0 (0.0)	0 (0.0)
Dementia	0 (0.0)	0 (0.0)	38 (97.4)	15 (100.0)
HDS‐R (0–30)^a^, mean scores	N/A	N/A	7.1	5.5
Hypertension	3 (9.4)	5 (16.1)	10 (25.6)	5 (33.3)
Cardiac disease	0 (0.0)	0 (0.0)	6 (15.4)	2 (13.3)
Chronic pulmonary disease	0 (0.0)	3 (9.7)	3 (7.7)	0 (0.0)
Renal disease	0 (0.0)	1 (3.2)	2 (5.1)	2 (13.3)
Cerebrovascular disease	0 (0.0)	0 (0.0)	11 (28.2)	4 (26.7)
Diabetes	1 (3.1)	3 (9.7)	9 (23.1)	3 (20.0)
Severity of COVID‐19^b^				
Asymtomatic	N/A	4 (12.9)	N/A	1 (6.7)
Mild	N/A	25 (80.7)	N/A	13 (86.6)
Severe	N/A	2 (6.4)	N/A	1 (6.7)
Time from vaccination to sample collection, mean days ± SD (range)				
Scheduled 21 days after the second dose	21.2 ± 1.40 (18–24)	20.8 ± 1.35 (18–23)	19.0 ± 0.76 (18–20)	19.6 ± 0.51 (19–20)
Scheduled 6 months after the second dose	184.3 ± 3.94 (179–193)	183.0 ± 3.41 (179–190)	175.5 ± 2.34 (174–181)	176.7 ± 1.87 (174–181)
Scheduled 21 days after the third dose	21.0 ± 1.11 (19–23)	20.6 ± 1.14 (18–24)	20.2 ± 1.32 (15–21)	20.4 ± 0.51 (20–21)
Time between the second and third doses, mean days ± SD (range)	230.4 ± 4.91 (223–240)	230.3 ± 4.39 (223–240)	215.4 ± 8.3 (203–231)	212.8 ± 7.1 (204–226)

*Note*: Data are no. (%) of individuals, unless indicated otherwise.

Abbreviations: HCW, healthcare worker; HDS‐R, the revised Hasegawa's dementia scale; N/A, not applicable; NHR, nursing home resident; SARS‐CoV‐2, severe acute respiratory syndrome coronavirus 2.

^a^ HDS‐R is a screening test for age‐associated dementia. Dementia was defined as a score of HDS‐R ≤ 20 (total maximum score of 30).

^b^ The definition of disease severity is based on reference no. 12.

### 2.2 Serological testing

Serological testing was performed using the collected serum samples. The quantitative levels of IgG antibodies for the spike (receptor‐binding domain [RBD]) and nucleocapsid antigens of SARS‐CoV‐2 were examined using the Abbott Architect immunoassays (SARS‐CoV‐2 IgG II Quant and SARS‐CoV‐2 IgG, Abbott, Park, IL, USA) according to the manufacturer's protocol. The anti‐RBD IgG levels of ≥4160 AU/mL were used as a surrogate marker of highly effective antibody neutralization, based on the manufacturer's instruction. This threshold corresponded to a 0.95 probability of obtaining a plaque reduction neutralization test (PRNT) ID_50_ at a 1:250 dilution. If the anti‐nucleocapsid antibody test using samples from SARS‐CoV‐2‐naive individuals revealed positive results, the individuals were re‐categorized as previously infected. A signal‐to‐cutoff value of ≥1.4 indicated positive detection of anti‐nucleocapsid antibodies.

### 2.3 Statistical analysis

Categorical variables were analyzed using Fisher's exact test. Continuous variables were compared using the Wilcoxon rank‐sum test. The association between two variables was analyzed using Spearman's rank correlation test. *P* values of <0.05 were considered statistically significant. All statistical analyses were performed using JMP Pro, version 14 (SAS Institute, Inc., Cary, NC, USA).

## 3 RESULTS

A total of 117 individuals, including 63 healthcare workers (mean age, 45.8 years; 32 SARS‐CoV‐2‐naive and 31 previously infected) and 54 nursing home residents (mean age, 83.8 years; 39 SARS‐CoV‐2‐naive and 15 previously infected), were eligible for the study. Based on anti‐nucleocapsid antibody testing, none of the SARS‐CoV‐2‐naive healthcare workers and residents were re‐categorized as previously infected. The baseline clinical characteristics of the 117 individuals are shown in Table 1. The number of participants decreased by 9 from our previous study, due to staff turnover and the transfer or death of residents. No individuals received immunosuppressant treatment, except for a previously infected healthcare worker with corticosteroid treatment. There was no difference in the distribution of clinical severity at the time of SARS‐CoV‐2 infection between previously infected healthcare workers and residents. All durations from vaccination to sample collection, including the scheduled 21 days after the second and third doses and the scheduled 180 days after the second dose, were similar among the healthcare workers and residents. The periods between the second and third doses were also similar, at approximately seven to seven and a half months.

**FIGURE 1:**
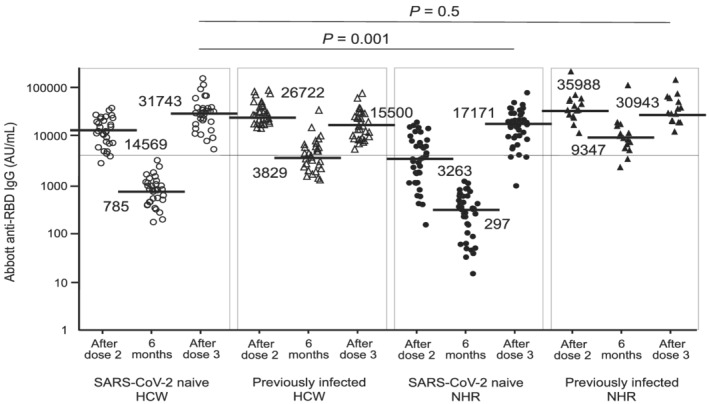
Anti‐spike (RBD) IgG antibody levels shortly after the second dose, 6 months after the second dose, and shortly after the third dose of BNT162b2 mRNA vaccination in SARS‐CoV‐2‐naive and previously infected healthcare workers and nursing home residents. The white circles represent data of SARS‐CoV‐2‐naive healthcare workers (n = 32). The white triangles represent data of previously infected healthcare workers (n = 31). The black circles represent data of SARS‐CoV‐2‐naive residents (n = 39). The black triangles represent data of previously infected residents (n = 15). After, doses 2 and 3 indicate 21 days after each dose. Six months indicate 6 months after the second dose. The horizontal solid bars and numbers in each group represent the median values. The horizontal line represents the value of 4160 AU/mL, a threshold level indicating highly effective antibody neutralization. RBD, receptor‐binding domain; SARS‐CoV‐2, severe acute respiratory syndrome coronavirus 2; HCW, healthcare workers; NHR, nursing home residents

Figure 1 shows the anti‐RBD IgG antibody levels shortly and 6 months after the second dose and shortly after the third dose. The median IgG level in SARS‐CoV‐2‐naive residents 6 months after the second dose was approximately two‐fold lower than that in SARS‐CoV‐2‐naive healthcare workers, with the lowest levels among the four groups. The median IgG level in SARS‐CoV‐2‐naive residents after the third dose was lower than that in SARS‐CoV‐2‐naive healthcare workers (17 171 vs. 31 743 AU/mL, *P* = 0.001); however, the IgG ratio of SARS‐CoV‐2‐naive residents to healthcare workers after the second and third doses changed approximately from one‐fifth (20%) to one‐half (50%). The frequency of IgG levels of ≥4160 AU/mL in the SARS‐CoV‐2‐naive residents (89.7%, 35/39) after the third dose was significantly higher than that after the second dose (41.0%, 16/39) (*P* < 0.0001). IgG levels in previously infected residents after the third dose were comparable with those in SARS‐CoV‐2‐naive healthcare workers after the third dose. However, an increase in antibody levels between the second and third doses was not observed in previously infected residents as well as previously infected healthcare workers, although a definite increase before and after the third dose was observed in both groups. Basic data on the antibody levels are shown in Table S1.

**FIGURE 2:**
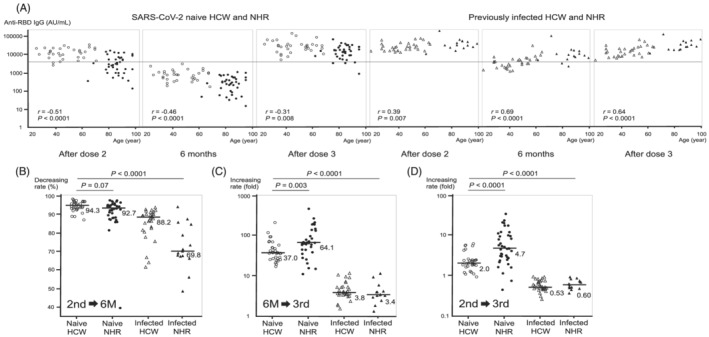
(A) Association of age with anti‐spike (RBD) IgG antibody levels after BNT162b2 mRNA vaccination. The white circles represent data of SARS‐CoV‐2‐naive healthcare workers. The white triangles represent data of previously infected healthcare workers. The black circles represent data of SARS‐CoV‐2‐naive nursing home residents. The black triangles represent data of previously infected residents. The horizontal line represents the value of 4160 AU/mL. (B) Decreasing rate of anti‐spike (RBD) IgG antibody levels at 6 months after the second dose of BNT162b2 mRNA vaccination. The rates (%) of reduction in the IgG levels between the two points of 21 days and 6 months after the second dose are shown. The horizontal solid bars and numbers in each group represent the median values. (C) Increasing rate of anti‐spike (RBD) IgG antibody levels before and after the third dose of BNT162b2 mRNA vaccination. The fold rates of increase in the IgG levels between the two points of 6 months after the second dose and 21 days after the third dose are shown. The horizontal solid bars and numbers in each group represent the median values. (D) Increasing rate of anti‐spike (RBD) IgG antibody levels between the second and third doses of BNT162b2 mRNA vaccination. The fold rates of change in the IgG levels between the two points of 21 days after the second and third doses are shown. The horizontal solid bars and numbers in each group represent the median values. RBD, receptor‐binding domain; SARS‐CoV‐2, severe acute respiratory syndrome coronavirus 2; HCW, healthcare workers; NHR, nursing home residents

The relationship between age and post‐vaccination anti‐RBD IgG levels is shown in Figure 2A. In SARS‐CoV‐2‐naive healthcare workers and residents, increasing age significantly correlated with a decrease in IgG levels at all points after vaccination, although the age‐related reduction decelerated after the third dose. In previously infected healthcare workers and residents, no decline in antibody levels with increasing age was observed after the third dose as well as the second dose. The decreasing and increasing rates of IgG levels between the points of sample collection were calculated for each individual in the four groups (Figure 2B–D and Table S1). The median decreasing rate at 6 months after the second dose in SARS‐CoV‐2‐naive residents was comparable with that in SARS‐CoV‐2‐naive healthcare workers, with a rate of over 90% (Figure 2B). The median rate of increase before and after the third dose in SARS‐CoV‐2‐naive residents was significantly higher than that in SARS‐CoV‐2‐naive healthcare workers (64.1‐ vs. 37.0‐fold, *P* = 0.003), and the median rate of increase in previously infected residents, similar to that in previously infected healthcare workers, was approximately three‐ to fourfold increase (Figure 2C). Finally, the rate of increase between the second and third doses was significantly higher in SARS‐CoV‐2‐naive residents than in SARS‐CoV‐2‐naive healthcare workers (4.7‐ vs. 2.0‐fold in median value, *P* < 0.0001) (Figure 2D). In contrast, IgG levels in previously infected healthcare workers and residents were reduced by approximately half after the third dose compared with the second dose.

## 4 DISCUSSION

In this study, we showed that anti‐spike (RBD) antibody levels of SARS‐CoV‐2‐naive nursing home residents were the lowest among all the groups examined at 6 months of post‐vaccination, with a high rate of reduction. Thus, 6 months after the initial vaccination series, SARS‐CoV‐2‐naive residents would have become much more vulnerable to breakthrough infection than the healthcare workers. In contrast, the antibody responses induced by the third (booster) vaccination in SARS‐CoV‐2‐naive residents are noteworthy. The increase in antibody levels between the pre‐ and post‐third doses was markedly higher than that in healthcare workers. This finding of our study agrees with the findings of recent studies that identified a robust increase in antibody levels after booster vaccination in nursing home residents and aged healthcare workers.^13^, ^14^ The mechanism of the robust antibody response in SARS‐CoV‐2‐native residents is unclear; however, it seems certain that the ability to induce a sufficient response to repeated antigen exposure persists in these residents, even though the primary response was reduced in them. Furthermore, our results suggest that residual SARS‐CoV‐2‐specific memory function induced robust antibody responses after the third dose in nursing home residents, even if their antibody levels were extremely low 6 months after the second dose. This implies that nursing home residents, poor responders in the initial SARS‐CoV‐2 vaccine program, could obtain sufficient antibody responses only by the additional booster dose. Our data indicate the requirement of a high application order of re‐vaccination for nursing home residents and the potential necessity of an earlier booster dose after the second or the initial three‐dose series.

Our previous study reported that antibody levels comparable with healthcare workers were induced by the initial vaccine series in previously infected residents, in contrast to SARS‐CoV‐2‐naive residents.^12^ In this study, the antibody levels of previously infected residents after the third dose were maintained at a level similar to healthcare workers. Thus, this finding suggests that in the case of previously infected residents, increasing age did not affect their antibody levels even at the third dose more than 6 months after the second. Instead, antibody levels after the third dose in previously infected individuals appeared to increase with age, similar to the observed response after the second dose.^12^ Notably, the antibody responses elicited by the third dose were poor in previously infected individuals (regardless of age), in contrast to SARS‐CoV‐2‐naive individuals. This finding was completely different from the rapid and robust responses observed after the first dose.^12^ We cannot accurately elucidate the underlying mechanism based on this finding, but we speculate that this finding could be attributed to exposure to the same antigen repeatedly in previously infected individuals compared to in SARS‐CoV‐2‐naive individuals. Further investigations are required to address this issue. However, booster vaccination would benefit previously infected individuals, considering the definite increase in antibody levels before and after the third dose. A recent report has shown significant vaccine effect against SARS‐CoV‐2 re‐infection among previously infected individuals.^15^ This suggests that high antibody levels acquired by vaccination are potentially useful for protecting against SARS‐CoV‐2 variant infection, not only in SARS‐CoV‐2‐naive individuals but also in those previously infected.

Abbott anti‐spike (RBD) IgG levels of ≥4160 AU/mL, which were used as a threshold of highly efficient antibody neutralization, did not necessarily reflect the standard levels required to protect against clinical SARS‐CoV‐2 infection. In our study, approximately 95% of SARS‐CoV‐2‐naive healthcare workers acquired antibody levels over 4160 AU/mL after the second dose. This finding is reasonable, considering the initial vaccine efficacy before the appearance of variants of concern. On the other hand, antibody levels required to protect against current SARS‐CoV‐2 variant infection may exceed the threshold, because current vaccines are derived from wild strains. The circulating omicron variant has been shown to significantly change its antigenicity compared to the wild or previous variant types.^16^ Therefore, the protective antibody levels for omicrons could be presumed to be higher than the threshold. The vaccine efficacy of the third (booster) dose against the omicron variant seems to be secured to some extent.^7^ Fortunately, higher antibody levels after the third dose than after the second dose may contribute to protective immunity against omicron variant infections. Further observations are needed regarding how much antibody levels after the third vaccination could result in a breakthrough infection with circulating variant viruses. The fourth vaccination has been initiated in several countries, including Japan. We currently plan to evaluate the kinetics of antibody levels before and after the fourth dose. In future investigations, it would be significant to evaluate whether wild‐type derived vaccine efficacy is still maintained for infections caused by omicron variants, including BA.5 subvariants.

A limitation of our study was the small sample size. Because the same participants were followed up in the cohort study, the number of participants decreased from the previous study.^12^ Based on its relatively small sample size, the conclusions and generalizability of this study may be limited. In addition, we were not able to determine the association of antibody levels with the underlying disease or frailty score, because of the difficulty in obtaining accurate data that reflected the participant's current situation.

In conclusion, SARS‐CoV‐2‐naive nursing home residents achieved sufficient antibody responses to the third (booster) vaccination, even though it had been over 6 months after the second dose. Previously infected residents, similar to previously infected healthcare workers, obtained a limited antibody response to the booster dose, although their antibody levels were comparable to those of SARS‐CoV‐2‐naive individuals. We have already experienced a severely affected and deteriorating situation due to COVID‐19 outbreaks in a nursing facility. Maintaining effective infection control measures for COVID‐19 outbreaks in nursing facilities is an important public health issue. We believe that our serological data from nursing home residents could be of significance for healthcare workers and future infection control measures.

## CONFLICT OF INTEREST

The authors have no conflict of interest to disclose.

## AUTHOR CONTRIBUTIONS

**Yong Chong:** Conceptualization; data curation; formal analysis; investigation; methodology. **Takeyuki Goto:** Investigation; methodology. **Naoki Tani:** Investigation; methodology. **Akiko Yonekawa:** Data curation. **Hideyuki Ikematsu:** Conceptualization; supervision; validation. **Nobuyuki Shimono:** Supervision; validation. **Yosuke Tanaka:** Conceptualization; supervision; validation. **Koichi Akashi:** Project administration.

### PEER REVIEW

The peer review history for this article is available at https://publons.com/publon/10.1111/irv.13030.

## Supporting information


**Table S1.** Basic data of anti‐spike antibody levels after BNT162b2 mRNA vaccination

## Data Availability

The data that support the findings of this study are available from the corresponding author on reasonable request.
